# Distribution Patterns of Essential Oil Terpenes in Native and Invasive *Solidago* Species and Their Comparative Assessment

**DOI:** 10.3390/plants11091159

**Published:** 2022-04-25

**Authors:** Jolita Radušienė, Birutė Karpavičienė, Mindaugas Marksa, Liudas Ivanauskas, Lina Raudonė

**Affiliations:** 1 Laboratory of Economic Botany, Nature Research Center, Akademijos Str. 2, 08412 Vilnius, Lithuania; joilta.radusiene@gamtc.lt (J.R.); birute.karpaviciene@gamtc.lt (B.K.); 2 Department of Analytical and Toxicological Chemistry, Lithuanian University of Health Sciences, Sukileliu Av. 13, 50162 Kaunas, Lithuania; mindaugas.marksa@lsmuni.lt (M.M.); liudas.ivanauskas@lsmuni.lt (L.I.); 3 Department of Pharmacognosy, Lithuanian University of Health Sciences, Sukileliu Av. 13, 50162 Kaunas, Lithuania; 4 Laboratory of Biopharmaceutical Research, Institute of Pharmaceutical Technologies, Lithuanian University of Health Sciences, Sukileliu Av. 13, 50162 Kaunas, Lithuania

**Keywords:** invasive species, distinctive terpenes, interspecific diversity, *Solidago × niederederi*, underutilized resources

## Abstract

The importance of invasive *Solidago* L. species to the environment creates a new approach to controlling their spread through the use of potentially high value raw materials. The aim of this study was to assess the distribution patterns of volatile compounds in the four *Solidago* spp., by identifying common and species-specific compounds with their potentials, and to confirm the origin of the spontaneous hybrid *Solidago × niederederi* on the basis of comparative assessment of essential oil (EO) profiles. Plant material in the flowering phase was collected in mixed populations from six different sites. The EOs were isolated separately from the leaf and the inflorescence samples by hydrodistillation for 3 h. The chemical analysis was performed by gas chromatography—mass spectrometry. Multivariate data analysis was employed to explain the interspecies relationships among *Solidago* spp. The results revealed the similarity among *Solidago* spp. EO profiles, which were dominated by monoterpenes and oxygenated compound fractions. *Solidago* spp. differed in species distinctive terpenes and their distribution between accessions and plant parts. Volatile compound patterns confirmed the origin of *Solidago × niederederi* between *Solidago canadensis* and *Solidago virgaurea*, with the higher contribution of alien species than native ones. Correct taxonomic identification of species is highly essential for the targeted collection of raw material from the wild for different applications. *Solidago* spp. can be considered to be underutilized sources of bioactive secondary metabolites.

## 1. Introduction

The use of plant products has grown remarkably in recent years, and research into natural products such as volatile terpenoids has become an important task for future human and animal well-being [1]. Natural products are generally easy to prepare and, due to their natural origin, are environmentally friendly and not financially challenging. In this respect, invasive species are of growing interest as a potential resource for obtaining high value-added products. *Solidago canadensis* L. (Canadian goldenrod) and *Solidago gigantea* Aiton (Giant goldenrod), native to North America, are considered to be one of the most aggressive plant invaders, which were introduced to Europe as garden ornamentals in the middle of the 18th century and began to spread in the 19th century [2]. Abandoned previously cultivated and disturbed areas contribute to a rapid and successful invasion of goldenrods. Both species form pure dense stands due to the clonal growth of their re-sprouting rhizome system, which provides a strong competitive ability to eliminate other grassland species while reducing biodiversity [3]. Propagation of the *Solidago* species easily by wind distributed seeds guarantees their distance dispersal and the occupation of new disturbed areas creating homogenized landscape [4]. On the other hand, spontaneous hybridization gives rise to new hybrids, such as sexually reproducing *Solidago × niederederi* Khek., which was first recorded in Austria more than a century ago [5]. The hybrid has been described as a new alien species between the invasive *S. canadensis* and native *Solidago virgaurea* L. (European goldenrod), which is spreading rapidly along with the parental species, increasing the negative impact on the native flora [6,7]. In addition, phytotoxicity of alien species has been often invoked as a significant factor negatively influencing the native species and composition of plant communities [8,9]. The fastest and cheapest result to eradicate or limit the invasion of goldenrods is the use of herbicides [10]. However, the application of herbicides has a negative impact on the environment and their use is limited. Based on the importance of goldenrods to the surrounding environment, a new approach has recently been developed so that invasive species can be a potential source of high value-added products, instead of eliminating them by labor consumption and environmentally unfriendly ways [11]. The high biomass produced by exotic goldenrods is a promising source of renewable energy that can be exploited in rural households as an alternative to expensive firewood and that do not compete with crops for food or animal feed [12]. Late-flowering goldenrods attract pollinators and are honey bee plants that are considered to be superior to crops treated with pesticides [13]. *Solidaginis virgaureae herba* is included in the ESCOP Monographs with therapeutic indications for the treatment of urinary tract and genital disorders [14]. The European Medicines Agency in a finalized community monograph of *Solidago virgaurea* confirms traditional use of this plant material for the treatment of minor urinary tract complaints [15]. Furthermore, *S. gigantea*, *S. canadensis*, and their hybrids, as well as *S. virgaurea,* are included in European Pharmacopoeia [16]. A wide range of specialized metabolites have been reported in goldenrod raw materials, of which phenolic compounds and EOs were considered to be the most valuable [17,18,19]. The comparative evaluation of phenolic compounds in *Solidago* spp. has been presented in our previous studies [20].

Essential oils (EOs), due to the structural diversity in their constituents, expose a wide range of biological effects and are of great interest as a source of functional ingredients for agriculture, food, cosmetics, and pharmaceuticals [21]. Numerous studies have reported the potential use of EOs for integrated weed and pest control as an environmentally friendly approach [22]. Significant antifungal activity of *S. canadensis* EO was found against *Botrytis cinerea*, which reduced fruit rot and successfully controlled gray mold in inoculated strawberries [21]. Elshafie et al. [23] has also demonstrated antimicrobial activities of *S. canadensis* EO in vitro against some other phytopathogens. In this way, EOs of alien *Solidago* spp. are a promising source for the development of organic pesticides and can meet the high demand for their production. In addition, the allelopathic activity of *S. virgaurea* is considered as the herbicidal potential of EOs for environmentally acceptable weed control in organic farming [24]. The EO of *S. canadensis* has been shown to exhibit significant cytotoxic and antiproliferative activities against human tumor cell lines, correlating with the terpene compounds [25,26].

The major constituents of EO usually contribute to the principal role of the biological activity of the mixture, so their efficiency can be predicted to some extent from the complex of components [27]. On the other hand, even minor components have been shown to play a significant role in different biological activities due to their complementary and synergistic effects [28]. Consequently, it is important to know the distribution of phytochemical compounds in species and their populations due to the targeted selection of raw materials for their possible multifunctional use. The study hypothesized that the screening of *Solidago* spp. growing in the same area and in their mixed populations could provide reasonable comparative information on the volatile profiles and their chemotaxonomic relationships and confirm the origin of the *S. × niederederi* taxon. The objectives of the study were: (1) to assess the distribution of volatile constituents in the populations of native and alien *Solidago* spp.; (2) to identify intraspecific and interspecific diversity in *Solidago* spp.; (3) to identify common and species distinctive volatiles; and (4) to confirm the origin of the spontaneous hybrid *S. × niederederi* on the basis of a multivariate comparative analysis of volatile profiles.

## 2. Results

### 2.1. Essential Oil Content of Solidago Species

Inflorescences took priority over the leaves in EO content in all the evaluated *Solidago* species. The highest yield of EO was obtained from inflorescences of *S. canadensis* (0.19–0.26%), followed by *S. gigantea* (0.16–0.23%), *S. × niederederi* (0.14–0.20%), and *S. virgaurea* (0.15–0.18%). Meanwhile, the leaves of *S. gigantea* accumulated the highest EO content (0.16–0.20%), followed by *S. canadensis* (0.14–0.18%), *S. × niederederi* (0.13–0.15%), and *S. virgaurea* (0.10–0.15%). Previous studies reported the similar range of EO yield in *S. canadensis* (0.18–0.27%) [23,29] and *S. gigantea* (0.15–0.16%) [29]. Kalemba [30] reported higher levels of EO contents in *S. virgaurea* (0.32–0.38%) than levels found in this study.

### 2.2. Chemical Profiles of Essential Oils

**Figure 1:**
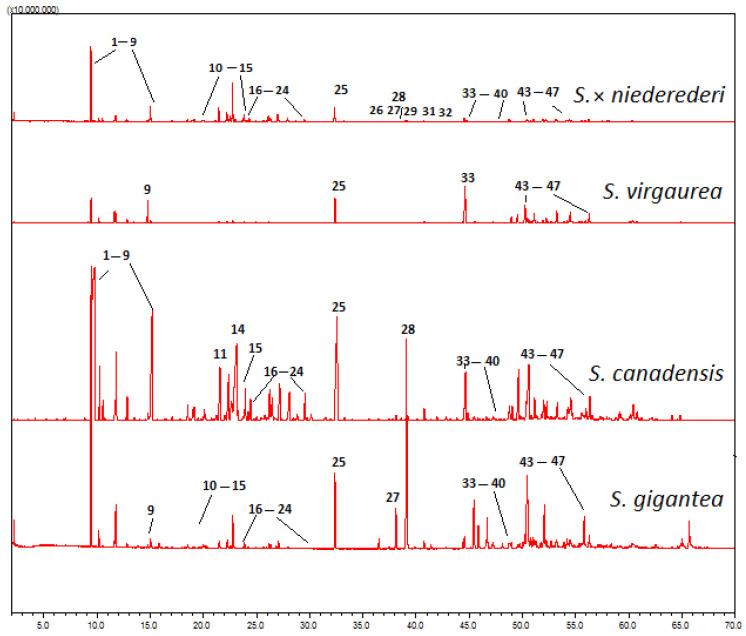
Examples of fingerprint profiles for *S. × niederederi*, *S. virgaurea*, *S. canadensis*, and *S. gigantea* inflorescence EOs performed by GC–MS using the GC–MS-QP2010 Ultra Gas system. The peak numbers correspond to the number of EO compounds listed in the Table 1.

The EOs of four *Solidago* spp. were dominated by monoterpenes, with an average of 43.9–74.6% of the total composition of EOs in inflorescences and 39.7–69.1% in leaves. The mean percentage of sesquiterpenes ranged from 15.0 to 35.4% in inflorescences and from 25.5 to 38.2% in leaf EOs. Chemical profiles for EOs of *Solidago* spp. varied according to the contents of individual compounds and their distribution among accessions and plant parts. Examples of fingerprint profiles for inflorescence EOs are shown in Figure 1.

#### 2.2.1. *Solidago gigantea*

Inflorescence EOs were dominated by monoterpene hydrocarbons (25.4%) and oxygenated sesquiterpenes (19.7%) followed by oxygenated monoterpenes (18.5%) and sesquiterpene hydrocarbons (15.3%). Meanwhile, oxygenated sesquiterpenes (23.3%) and oxygenated monoterpenes (23.1%) were the major chemical fractions in leaf EOs.

The principal compounds in all inflorescence and leaf EOs were *α*-pinene, bornyl acetate, spathulenol, isospathulenol, and caryophyllene oxide. Nevertheless, there were high differences for other compound prevalences and their concentrations between samples and plant parts. Germacrene D was a major common component in inflorescence oils (3.4–18.3%), whereas this compound was detected in less than half of the leaf samples (1.6–12.7%). One of the major components in the inflorescences was *o*-cymene (6.8–18.4%), while among the principal compounds in the leaves was *β*-cubebene (17.3–19.6%), but these compounds were found in only a few EO samples. Additionally, *trans*-pinocarveol (0.6–1.1%), *cis*-verbenol (0.3–1.0%), *trans*-verbenol (1.2–3.0%), and *γ*-muurolene (0.5–1.1%) were found in minor concentrations in all inflorescence EOs. Meanwhile, camphene (1.54–3.9%), *β*-pinene (1.0–3.4%), *o*-cymene (0.8–4.9%), limonene (0.6–1.3%), and *γ*-cadinene (0.6–1.5%) were common in all leaf EOs.

In agreement with our results, *α*-pinene, bornyl acetate, germacrene D, and spathulenol have been previously reported to be major components of *S. gigantea* EOs [29,31,32,33]. In addition, cyclocolorenone, *α*- and *γ*-gurjunene, khusinol, and/or ledol and selina-3,11-dien-6-α-ol were also reported as the predominant constituents [31,33], although these sesquiterpenoids were not detected in the *S. gigantea* EOs tested. According to Gruľová et al. [34], *S. gigantea* EOs were dominated by sesquiterpene hydrocarbons such as *δ*-cadinene, *γ*-muurolene, *α*-cubebene, and *γ*-cadinene, and two of them, *γ*-muurolene and *γ-*cadinene, were common in the inflorescence or leaf EOs studied.

#### 2.2.2. *Solidago canadensis*

Monoterpenoids were predominant in *S. canadensis* EOs, with an average of 46.6.0% in inflorescences and 26.4% in leaves, followed by monoterpenes (28.0%) in inflorescences and sesquiterpenes (24.9%) in leaves. The first or second principal components in most of inflorescence EOs were *α*-pinene (0.1–36.1), *trans*-verbenol (5.2–21.7%), and bornyl acetate (3.8–19.8%). Among the most abundant constituents in leaf and inflorescence EOs were *α*-pinene (1.3–21.8) and bornyl acetate (6.5–20.4%). Leaf EOs were dominated by sesquiterpene hydrocarbons as *β*-cubebene (6.1–33.6%) and germacrene D (4.0–45.2%), and their concentrations exceeded those in inflorescence oils (0.8–13.1% and 1.9–11.4%, respectively). The other main constituent common for all leaf EOs was isospathulenol (0.7–10.2%). In addition, carvacrol was present as a major compound in two leaf EOs (22.83 and 23.7%) and was not detected in the remaining samples. Other components with noteworthy values in both inflorescence and leaf EOs were limonene (0.3–16.2% and 0.8–10.9%, respectively) and caryophyllene oxide (1.6–10.0% and 1.4–10.4%, respectively). The data revealed that *cis*-verbenol (0.6–3.7%), pinocarvone (0.9–2.7%), myrtenal (0.8–3.4%), and verbenone (1.0–5.2%) were detected in all inflorescence EOs in highly variable concentrations. Meanwhile, camphene (0.4–2.2%), *β*-pinene (0.7–2.7%), *β*-caryophyllene (0.8–6.9%), *trans*-verbenol (0.5–5.4%), *β*-elemene (0.6–5.9%), epoxyazulene (0.7–6.47%), and spathulenol (0.4–3.8%) were found in all or most of the leaf EOs.

The presented results are in agreement with previous studies that confirmed *α*-pinene, limonene, bornyl acetate, germacrene D, *β*-cubebene, and caryophyllene oxide among the predominant compounds in *S. canadensis* EOs [19,23,25,33,35]. In addition, studies from different countries have shown that *γ*-cadinene and myrcene [36,37] sabinene [36], cyclocolorenone [29], or thymol [34] were among the major compounds in *S. canadensis* EOs. Meanwhile, our results showed a low concentration or frequency of these compounds in the samples tested.

#### 2.2.3. *Solidago × niederederi*

*Solidago × niederederi* inflorescence and leaf EO profiles were characterized by oxygenated monoterpenes (41.5 and 35.7%, respectively), followed by monoterpenes (26.4 and 17.7%, respectively), sesquiterpenoids (17.0 and 16.9%, respectively), and sesquiterpenes (6.0 and 15.0%, respectively). The principal constituents in the inflorescence EOs were *α*-pinene and *trans*-verbenol, which were the first or second major compounds in seven EOs, accounting for 22.3–31.7% and 13.4–22.9%, respectively. Caryophyllene oxide was among the major constituents in four (7.9–25.3%) and bornyl acetate in three (12.8–21.7%) EOs. Meanwhile, limonene and humulene epoxide II were present in most of the samples, however, dominated only in two (12.2 and 16.3%) and one (28.4%) samples, respectively. All inflorescence EOs contained varied levels of *α*-campholenal (0.9–6.2%), *trans*-pinocarveol (1.6–5.4%), and verbenone (0.6–5.1%). Other compounds with a mean content of 1 to 5.0% were *β*-pinene, camphene, *cis*-verbenol, pinocarvone, myrtenal, myrtenol, *trans*-carveol, germacrene D, epoxyazulene, and spathulenol.

*Solidago × niederederi* leaf EOs contained seven compounds in concentrations above 10% in at least one sample and were considered as principal compounds. The major compounds such as *α*-pinene (1.1–25.8%), *trans*-verbenol (2.2–20.2%), bornyl acetate (4.2–20.2%), and caryophyllene oxide (2.1–38.1%) were found in all leaves in highly different concentrations. Verbenone and germacrene D were present in most EOs, averaging 5.2 and 8.8%, respectively, with the exception of the two EOs in which these compounds were predominant, accounting for 20.2 and 18.0%, respectively. Meanwhile, *β*-cubebene was detected only in two oils (16.4 and 23.9%) in which it was the first or second major compound. One leaf EO contained noteworthy concentrations of thymol (6.4%) and carvacrol (9.5%). In addition, 21 compounds were detected with a mean content of 1 to 5.0%, the most prominent of which were sesquiterpenes such as *β*-copaene, *β*-bisabolene, epoxyazulene, humulene epoxide II, and isospathulenol. Similar results for the composition of the major compounds in *S. × niederederi* EO have been recently published [26]. However, the results of only one plant accession were reported, making the comparison insufficient as the prevalence and number of compounds varied among samples.

#### 2.2.4. *Solidago virgaurea*

*Solidago virgaurea* inflorescence and leaf EOs were dominated by monoterpenes fraction, which accounted for an average of 35.9 and 49.0% of the total EO composition, respectively, followed by sesquiterpenes (20.7 and 16.9%, respectively). The main compounds in inflorescence EOs were *α*-pinene (18.8–36.3%), *β*-copaene (5.3–21.2%), and caryophyllene oxide (6.7–11.4%). All leaves were predominated by trans-verbenol (10.2–49.0%), two samples prevailed by *α*-pinene (22.4 and 23.3%) and caryophyllene oxide (10.1 and 14.7%), and one by verbenone (16.9%). Inflorescence and leaf EOs contained 22 and 29 compounds, respectively, with a mean percentage greater than 1% and less or equal to 5%. Among them, the most prominent were *β*-pinene, limonene, verbenone, *α*-campholenal, *trans*-pinocarveol, pinocarvone, bornyl acetate, *α*-copaene, germacrene D, cubebol, *α*-muurolene, *δ*-cadinene, and spathulenol, which were common to all inflorescence and/or leaf EOs.

Similar to our identification, previous studies have confirmed the dominance of monoterpene and sesquiterpene fractions in *S. virgaurea* EOs [30,32]. Monoterpenes such as *α*-pinene, myrcene, *β*-pinene, and limonene together with sesquiterpene germacrene D have been reported as the major constituents in *S. virgaurea*. In addition, oxygenated sesquiterpenes, humulene epoxide II, spathulenol, selina-3,11-dien-6-α-ol, and caryophyllene oxide have been also considered as major compounds in *S. virgaurea* EOs [26,33]. Meanwhile, in this study, only *α*-pinene and caryophyllene oxide were found among the predominant compounds in all EOs tested, and the contents of the other mentioned compounds differed among samples. Meanwhile, selina-3,11-dien-6-α-ol was not detected at all in the presented EOs.

### 2.3. Interspecific Differences

The results presented revealed similarities and differences in the frequency of distribution and contents of EO constituents among the four *Solidago* spp. Significant differences in monoterpene and sesquiterpene fractions were found between the inflorescences of the species studied (Table 1). Meanwhile, the chemical groups of compounds in leaves did not differ significantly between the four *Solidago* spp. (Table 2).

Oxygenated monoterpenes predominated in the EOs of *S. canadensis* and *S. × niederederi* inflorescences and *S. virgaurea* and *S. × niederederi* leaves. The highest proportion of sesquiterpenoids among the species was found in *S. gigantea* inflorescences and leaves. Mono- and sesquiterpenes prevailed in the *S. virgaurea* inflorescence EOs; however, no significant differences were found between the species for monoterpenes. The common principal constituents of the inflorescence and leaf EOs of all four *Solidago* species were *α*-pinene, bornyl acetate, and caryophyllene oxide. Species showed significant differences (*p* ≤ 0.05) in the accumulation of bornyl acetate and caryophyllene oxide in leaf EOs, but no differences in *α*-pinene were observed between species (Table 1 and Table 2). The highest mean concentration of bornyl acetate was found in inflorescence and leaf EOs of both *S. gigantea* and *S. canadensis*, while caryophyllene oxide prevailed in *S. × niederederi* and *S. virgaurea*. The other major compound *trans*-verbenol was prevalent in all inflorescence EOs with the highest level (*p* ≤ 0.001) in *S. canadensis* and *S.* *× niederederi*. Meanwhile, the leaves of *S. virgaurea* and *S.* *× niederederi* had priority over the other two species in accumulation of trans-verbenol. Inflorescences of *S. gigantea* accumulated the highest (*p* ≤ 0.001) level of germacrene D compared to other species, but this compound did not differ significantly (*p* > 0.05) in the leaves between species. The inflorescences and leaves of *S. gigantea* were in priority to other species in accumulation of oxygenated sesquiterpenes as epoxyazulene, spathulenol, and isospathulenol. In addition, *γ*-muurolene, differently other species, was a common compound in all inflorescence EOs of *S. gigantea*, while other monoterpene hydrocarbons such as camphene and o-cymene were common in all leaf EOs of this species.

The inflorescence EOs of all species differed significantly in the mean concentrations of oxygenated monoterpenes, such as *α*-campholenal, *trans*-pinocarveol, *cis*-verbenol, pinocarvone, verbenone, and *trans*-carveol, with the highest levels and frequency found in *S. canadensis* and *S. × niederederi*, followed by *S. virgaurea*. The same compounds, with the exception of *trans*-carveol, differed significantly in leaf EOs, with the highest concentrations in *S. virgaurea* and *S. × niederederi* leaves. Meanwhile, *trans*-carveol was detected in small amounts only in *S. canadensis* and *S. × niederederi* leaves. In addition, *S. virgaurea* inflorescence EOs differed from other species in the highest levels and frequency of distribution in *α*- and *β*-copaene, cubebol, *α*-muurolene, and *δ*-cadinene. As a consequence, quantitative rather than qualitative differences were observed between the species EOs. However, 10 compounds common in more than 30% of all studied inflorescence and/or leaf EOs did not differ significantly between *Solidago* spp. (Table 1 and Table 2). Among them, the most abundant were *α*- and *β*-pinene, limonene, bornyl acetate, germacrene D, and caryophyllene oxide.

### 2.4. Principal Component Analysis (PCA)

**Figure 2:**
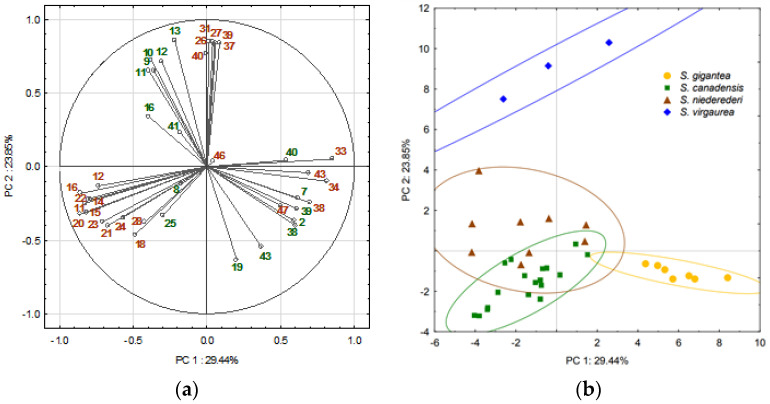
PCA model representing the accumulation of terpenes in inflorescences and leaves of four *Solidago* species: (**a**) loading plot of the variables contributing to PC1 and PC2; (**b**) scores plot for the tested EOs with a 95% ellipses confidence limit for each species. The numbers of the variables correspond to the numbers of EO compounds listed in the Table 1 and Table 2. Inflorescence variable numbers were marked in brown; leaf variable numbers were marked in green.

PCA was employed to explain the phytochemical relationships arising due to inter- and intraspecific differences between the four *Solidago* spp., using the selected EOs compounds. A scree plot criterion was applied to reduce the number of PCs for explaining the variance in the selected variables. A two-dimensional PCA square matrix model explained more than 53.3% of the total variance and was used to visualize the available patterns of *Solidago* spp. EOs profiles (Figure 2). PC3 explained only 6.8% of the total variance and had no significant effect on scores differentiation, so results were not presented. PC1 accounted for 29.4% of the total data set variance and showed high negative correlation with *α-*campholenal, *trans*-pinocarveol, *cis*- and *trans*-verbenol, pinocarvone, myrtenal, myrtenol, verbenone, and trans-carveol and positive with carvone, germacrene D, *γ*-muurolene, *γ*-cadinene, spathulenol, and isospathulenol in inflorescences, and camphene, *o*-cymene, epoxyazulene, and (*E*)-nerolidol in leaves (Figure 2a). PC2 explained 23.6% of the total data set variance and was highly associated with positive loadings of *α*- and *β*-copaene, *α*-cubebene, cubebol, *α*-muurolene, and *δ*-cadinene in inflorescences and with *α*-campholenal, *trans*-pinocarveol, *cis*-verbenol, and pinocarvone in leaves, as well as with negative loadings of bornyl acetate and isospathulenol in leaves.

The PCA score plot model showed the arrangement of 40 EOs into two separate and two overlapping ellipses, each with a 95% confidence interval limit (Figure 2b). The group on the right-hand plot combined all *S. gigantea* EOs along the positive PC1. Variables with high PC1 loadings contributed the highest impact on the grouping of *S. gigantea* samples were germacrene D, *γ*-cadinene, *γ*-muurolene, and spathulenol in inflorescences, and camphene, *o*-cymene, epoxyazulene, (*E*)-nerolidol, and spathulenol in leaves. These compounds were shared among all *S. gigantea* EOs in the highest amounts compared to the other species studied. Conversely, variables with high PC2 loadings had a weak contribution on the grouping of EOs and were found in minor quantities in *S. gigantea*.

*Solidago virgaurea* EOs were clustered into a separate group on the upper positive side of the score plot, in distance from all other samples, indicating differences in their composition. The location of the samples can be explained by the same position of variables, which have a significant positive contribution to PC2. Variables with unit vectors close to each other were positively correlated, and their impact on the position of the samples was similar. Thus, *α*- and *β*-copaene, *α*-cubebene, cubebol, *δ*-cadinene, and *α*-muurolene in inflorescences, and *α*-campholenal, *cis*-verbenol, *trans*-verbenol, pinocarvone, and *trans*-pinocarveol in leaves, were common in *S. virgaurea* EOs and found in significantly higher amounts than in other species analyzed. Meanwhile, *S. canadensis* and *S. × niederederi* EOs were clustered into two partially overlapping ellipses, mainly in the left-hand score plot, showing the similarity of the volatile compound patterns. The arrangement of inflorescence EOs for both species coincided with a significant correlation of *α*-campholenal, *trans*-pinocarveol, pinocarvone, verbenone, *cis*-verbenol, *trans*-verbenol, myrtenal with PC1. In addition, the clustering of *S. canadensis* was influenced by myrtenol, *trans*-carveol, and carvone. Variables with high PC2 loading had no significant impact on EOs’ arrangement, except for *trans*-verbenol in *S. × niederederi* leaves, showing similarity of this taxon to *S. virgaurea*. Meanwhile, a previous study of phenolic compounds showed greater chemical similarity of *S. × niederederi* to *S. virgaurea* than to *S. canadensis* [20]. Consequently, the phytochemical patterns complemented the evidence of *S. × niederederi* origin between native *S. virgaurea* and invasive *S. canadensis*, with the higher contribution of alien species than that of native ones. In addition, *S. × niederederi* EOs were much more scattered on the PCs space, indicating higher diversity than other species, suggesting that *S. × niederederi* is a continuously evolving taxon.

Consequently, *Solidago* spp. EOs differed significantly in the presence of terpenes that could be considered as species distinctive components. The inflorescence EOs of *S. gigantea* differed from other species by γ-cadinene, γ-muurolene, and spathulenol, and the leaves by camphene, *o*-cymene, epoxyazulene, (*E*)-nerolidol, and spathulenol. The inflorescences of *S. canadensis* and *S. × niederederi* differed significantly from the other species by the accumulation of oxygenated monoterpenes, such as *α*-campholenal, *trans*-pinocarveol, pinocarvone, verbenone, *cis*-verbenol, *trans*-verbenol, and myrtenal. In addition, *S. canadensis* inflorescence EOs were characterized by the prevalence of myrtenol, *trans*-carveol, and carvone, while *S. × niederederi* leaves were prominent by *trans*-verbenol. The species distinctive volatiles in *S. virgaurea* inflorescence EOs were *α*- and *β*-copaene, *α*-cubebene, cubebol, *δ*-cadinene, and *α*-muurolene, and in the leaves—*α*-campholenal, *cis*-verbenol, *trans*-verbenol, pinocarvone, and *trans*-pinocarveol. Consequently, multivariate data analysis allowed for an explanation in the intra- and interspecific diversity in four *Solidago* taxa according to the differences in EO volatiles.

## 3. Discussion

Alien goldenrods are morphologically and phylogenetically close to each other, but differ in their ploidy level; *S. gigantea* is tetraploid (2n = 36), while *S. canadensis,* and *S. × niederederi* together with native *S. virgaurea* are diploids (2n = 18) [3,38]. The close relationships between *Solidago* spp. were reflected in the similarity of their phytochemical profiles. A comprehensive metabolomics approach to the different species indicated that the successful alien species had higher total number and more unique composition of secondary metabolites than their native congeners [39]. A comparison of the current and previous reports showed that our results are in agreement with previous reports for higher proportions of sesquiterpenoids in *S. gigantea* and hydrodrocarbons in *S. virgaurea* EOs [26,29,32,33]. Similar to our identification, the most abundant common compounds detected in the present study were also observed in previous studies on different species. Thus, the volatiles commonly found in various plant species have a high potential to accumulate in *Solidago* spp. EOs as well. On the other hand, there were compounds such as thymol and carvacrol, sporadically high levels of which were found in only a few *S. × niederederi* and *S. canadensis* EOs. Populations rich in thymol and carvacrol, compounds with a broad spectrum of biological activity [40,41,42], can be considered as a source of high potential raw material. In addition, *Solidago* spp. EOs differed significantly in some of the terpenes that can be considered as volatiles with great potential in chemophenetic studies of species. The first comparative study on terpenes as species distinctive compounds confirmed the origin of *S. × niederederi* as an interspecific taxon between *S. canadensis* and *S. virgaurea*. According to Orians [43], parental phytochemicals in hybrids tend to mostly express as either intermediate or similar to one of the parent’s compositions. The composition of *S. × niederederi* EOs was close to *S. canadensis*, one of parental species. The chromosome number may provide information about the hybrid origin of the species when it display allopolyploidy, but *S. × niederederi* exhibit a homoploid condition compared to its parental species and may backcross toward parental species. In this way, hybridization can increase the invasive capacity of goldenrods through gene introgression and significantly alter the ecosystems in which they grow [44]. However, species–specific compounds and chromosome number are not the main tools for hybrid identification. DNA fingerprinting techniques are the most reliable tools, but the use of additional phytochemical markers can provide insight into the ecological performance of hybrids and their further applications [45].

More often, the lower proportion of monoterpenes compared to sesquiterpenes [46] accounted for a higher proportion in the *Solidago* spp. EOs tested. Oxygenated monoterpenes have been proven to be the main phytotoxic active compounds in different plant EOs and have been highlighted as predictors of potential bioherbicides [47]. In addition, a tendency has been suggested that the monoterpene-rich EOs to promote higher phytotoxicity than sesquiterpene-rich EOs [48]. Comparative studies on the toxic activity of oxygenated monoterpenes revealed that the most active were alcohols, myrtenol and *trans*-pinocarveol, and ketones, verbenone and pinocarvone, which can be classified as predictors of the herbicidal activity of EOs [49,50,51]. Thus, *S. canadensis* and *S. × niederederi*, whose EOs differed from other species in some of oxygenated monoterpenes, suggested the potential of their raw materials for herbicidal activity. Meanwhile, terpene hydrocarbons have been found to be low phytotoxic EOs compounds [40,52]. On the other hand, Lawson et al. [36] reported that monoterpenes such as α- and β-pinene, limonene, or myrcene showed weak antifungal activity. Recent findings reported that (*E*)-nerolidol and spathulenol, which were presented as species–specific sesquiterpenoids in *S. gigantea* EOs, revealed effective allelopathic and insecticidal effects with potential for the developing a new natural pesticide [53,54,55]. Many findings have demonstrated the biological activity of caryophyllene oxide [53,55,56,57,58] that was a common compound in the presented *Solidago* spp. EOs. The potential biological activity of EOs is associated with the presence of high oxygenated compounds, as confirmed by a systematic review of phytotoxicity studies [59]. In this context, *S. canadensis* and *S. × niederederi* EOs, are of greatest interest in the development of new and safe bioproducts.

Considering the previous and presented results for *Solidago* spp. EOs, the prevalence of predominant and other compounds varied across different studies, and their comparison is not informative enough. Reports often provide single sample data that are difficult to summarize as a species–specific composition of volatiles. According to Zidorn [60], correct taxonomic identification, geographical location, plant harvesting season, plant parts, and other indirect factors are crucial in phytochemical studies, which often receive little attention. Exogenous factors or environmental regulated factors such as light, precipitation, growing site and soil are often considered to be the most important factors modifying the qualitative/quantitative composition of EOs [61]. Experiments have shown that plants exposed to drought stress increased the concentration of monoterpenes to protect plant cells from ROS damage [62]. Meanwhile, Caser et al. [63] found that drought increased the production of sesquiterpenoids and decreased monoterpenoids. According to Paulsen and Selmar [64], increased terpene synthesis is not supported by carbon allocation theory, but is attributed to changes in biomass production. In addition, differences in the chemical profiles of EOs are often explained in the context of the interaction of the metabolic inversion of the ratio of oxygen-free to oxygen-containing terpenes with the surrounding environment. Sesquiterpenes have been observed to be predominate during the dry season, while higher concentrations of sesquiterpenoids were found during the wet season [65]. The water scarcity increased the production of monoterpenoids and monoterpenes, while the opposite trend was observed for sesquiterpenes [66]. On the other hand, Tsusaka et al. [67], investigating the influence of genetic and environmental factors on sesquiterpenoids in *Atractylodes lancea*, (Thunb.) DC. found that the genotype had a greater effect on EO compounds than the conditions of the plant cultivation year. The volatiles were stable despite the changing growing conditions, but the absolute values of terpenoids were induced by the site of cultivation. Tardugno et al. [68] determined that the composition of *Thymus vulgaris* L. essential oils were highly influenced by the cultivating techniques. Over time, local environment leads to differences in metabolomics and the formation of ecotypes and chemotypes within a species [69].

The intraspecific differences in *Solidago* spp. volatile compounds observed in the present study can be explained by genotypic differences, as the plants grew under close conditions. Sexual reproduction helps maintain a high level of genetic and phenotypic diversity in goldenrods. Our previous study showed a high morphological diversity in *Solidago* spp. both between populations and between individual genets [3]. Similarly, high variability in volatiles and morphological characters was observed in wild *Mentha longifolia* L. accessions growing in the same field [70]. According to Zhao et al. [71], high genetic variations are characteristic within invasive and native areas of *S. canadensis* populations. Considerations suggested that the study of local populations makes it possible to identify intraspecific diversity that potentially reflects local genetic changes rather than the controversial dependence of terpenes synthesis under changing environmental conditions. The outstanding diversity in the goldenrods studied allows the selection of accessions in terms of the desired composition of EO volatiles. Phytochemical profiling of plant raw materials is an informative tool to learn about their potential for further development of new natural products.

## 4. Material and Methods

### 4.1. Plant Material

Plant material of four *Solidago* spp. in the flowering phase was collected from six different sites in Vilnius district, Lithuania, in August 2018. Eighteen accessions of *S. canadensis*, seven of *S. gigantea*, nine of *S. × niederederi*, and three accessions of *S. virgaurea* were collected at least one kilometre apart from each other in abandoned dry grasslands and disturbed farmlands (Table 3). The vegetation of the collection sites was characterized as semi-ruderal dry grassland dominated by plant communities of *Agropyretea intermedii-repentis* and *Artemisietea vulgaris*. The EOs in habitats were sand or sandy loam, with low to moderate humus content (1.8–2.7%), rich in phosphorus (126–260 P_2_O_5_ mg kg^−1^) and potassium (146–205 K_2_O mg kg^−1^), pH_KCl_ varied from 5.8 to 7.1.

The harvested plant material consisted of shoots of a single genet derived from a single seed. Individual genets were identified by phenological and morphological characteristics and by rhizome connections. The accessions of the same species were collected at least five meters apart from each other if more than one accession was collected from the same site. The plant material was dissected into inflorescences and leaves and dried separately at 25 °C. The botanical identification of species was based on morphological diagnostic characters such as the shape and size of inflorescences and ray flowers, stem color, and stem hairiness by Birutė Karpavičienė and Jolita Radušienė [3]. The specimens of evaluated *Solidago* spp. were deposited in the Herbarium of the Institute of Botany of Nature Research Centre (BILAS), Vilnius, Lithuania.

### 4.2. Isolation of Essential Oils

The plant material from 30 g of air-dried leaves and inflorescences was hydrodistilled separately for three hours using a Clevenger type apparatus. Each sample of yellowish EO was dried over anhydrous sodium sulphate and stored in a sealed vial at 4 °C until analysis. A sample preparation for chemical analysis included 1.0 μL of EO added into 1.0 mL of *n*-hexane following previous studies [23,33]. The essential oil content was calculated as relative percentages per 100 g of dry plant material.

### 4.3. Analysis of Essential Oils

The EOs analysis was performed using the GCMS-QP 2010 Ultra system equipped with a Shimazu autoinjector AOC-5000 (Shimadzu, Europa GmbH). A capillary column RXi-5MS (30 m × 0.25 mm i.d. × 0.25 film thickness µm) (Restek, Bellefonte, PA, USA) was used. The sample injection volume was 1 µL, a split ratio was 1:60 (*v*:*v*) and the split injector temperature was 260 °C. Helium was used as carrier gas with flow rate of 1.22 mL min^−1^. The initial column temperature was 50 °C, held for 5 min and raised to 200 °C at the rate of 2 °C min^−1^, then raised from 200 to 315 °C at the rate of 15 °C min^−1^ and held for 5 min. The detector ion source and interface temperatures were 200 °C and 280 °C, respectively. Mass spectra were acquired at an ionization voltage of 70 eV, a scan rate of 2500 *m/z* within the range of 29–500 *m/z* and a scan time of 0.2 s. The chromatographic analysis was run in triplicate.

### 4.4. Identification and Quantification of Components

The linear retention indices (LRI) of compounds were calculated using a homologous series of *n*-alkanes C8–C26 (Sigma-Aldrich, UK, purity >99.2%) injected at the beginning of the analysis and comparing the retention times of the eluted peaks with those of the alkanes [72]. Chromatographic data were analyzed using GC–MS solution software (Shimadzu, Europa GmbH). The EO constituents were identified by comparing the unique mass spectral fragmentation patterns of each peak with the mass spectral computer library database and those presented as standards in the NIST 14, FFNSC, WR10, and WR10R libraries, as well as comparing the obtained LRI with presented in NIST 14 datasets and reference [73] corresponding to the conditions for dimethylsilicone stationary phase with 5% phenyl groups. The relative percentages of analytes as the mean of the three runs were calculated from their peak areas in the chromatographic profiles without the use of correction factors corresponding to the conditions for the stationary phase of dimethylsilicone with 5% phenyl groups.

The repeatability and intermediate precision of analysis, expressed as relative standard deviation (RSD), was evaluated by performing the retention time and peak area values of five analytes in the same *S. canadensis* EO extract for intra- and inter- daily tests (Table 4).

Repeatability was determined in five consecutive injections of EO in the same day. The RSD for the retention time ranged from 0.22 to 0.32% and for relative peak area from 0.33 to 0.45%. Intermediate precision was assessed by five injections over two different days, with RSD values ranging from 0.54 to 0.92% for retention time and 0.98 to 2.21% for peak areas. The accuracy of the quantification was satisfactory, as RSD value within the 3% range is generally considered as acceptable.

### 4.5. Data Analysis

Multivariate statistical analysis was performed using software package Statistica 10.0 (StatSoft Inc.). The Kruskal–Wallis ANOVA was used to determine the differences between species. Significant differences were specified by two-tailed test at *p* ≤ 0.05. Principal component analysis (PCA) was used to identify the similarities and differences between the EOs analyzed using statistically independent variables. The PCA was based on 24 inflorescence and 16-leaf standardized variables that different significantly between species and that represented constituents detected in 30% or more of EO samples in at least one species. Leaf and inflorescence data sets were pooled and used in PCA, resulting in more convincing results than separate leaf and inflorescence PCAs.

## 5. Conclusions

The frequency and percentage of distribution in the volatile constituents of *Solidago* spp. varied depending on the species, accessions, and plant parts. The principal compounds common to all *Solidago* spp. inflorescence and leaf EOs were *α*- and *β*-pinene, limonene, bornyl acetate, germacrene D, spathulenol, and caryophyllene oxide. *Solidago* spp. differed significantly in some of the distinctive terpenes that can be considered as compounds with high potential for chemophenetic and taxonomic studies of the genus. A comparison of volatile profiles for *Solidago* spp. confirmed the interspecific origin of *S. × niederederi* between *S. canadensis* and *S. virgaurea* with a higher metabolic contribution of alien species than native ones. The findings provide the bioprospecting of *Solidago* spp. as a source for specified composition of volatiles. The vast resources of invasive goldenrods are of great interest as a convenient and readily acceptable, underutilized source of natural bioactive compounds that can be used for different applications.

The combination of fingerprint and multivariate data analysis demonstrated a simplified assessment of the quality of wild plant materials. Correct species identification is essential for the development of raw material quality control protocols for the targeted collection and assessment of raw materials from wild populations. The screening of a relatively large number of plant accessions from mixed populations of different species allows for a more reasonable comparison of their volatile profiles and enables prediction of the most likely quality of raw materials harvested from the wild.

## Figures and Tables

**Table 1:** The frequency of distribution (F, %) of the compounds detected in more than 30% of the inflorescence EO samples in at least one of the four *Solidago* species; their mean relative percentage (M) with SD and significance (*p*) of interspecies differences according to the Kruskal–Wallis two-tailed test.

No	Compounds	LRI Exp.	LRI Ref.	*S. gigantea* (*n* = 7)			*S. canadensis* (*n* = 18)			*S. × niederederi* (*n* = 9)			*S. virgaurea* (*n* = 3)			*p*
				F	M	SD	F	M	SD	F	M	SD	F	M	SD	
1	*α*-pinene	930	930	100	10.7	8.31	100	18.1	13.84	100	17.4	12.84	100	28.6	8.96	0.196
2	camphene	945	948	71.4	1.8	0.77	72.2	1.7	0.69	77.8	1.2	0.34	100	1.0	0.35	0.713
3	thuja-2,4(10)-diene	950	957	28.6	0.6	0.35	66.7	0.5	0.16	44.4	0.7	0.32	66.7	0.6	0.13	0.504
4	sabinene	971	976	71.4	2.5	2.07	66.7	1.1	0.79	66.7	1.3	1.79	33.3	0.6	–	0.258
5	*β*-pinene	974	980	71.4	3.0	0.90	83.3	2.3	1.40	88.9	2.2	1.71	100	3.6	1.51	0.536
6	*β*-myrcene	992	991	42.9	1.6	0.34	55.6	0.8	0.60	33.3	0.6	0.15	66.7	1.5	0.23	0.564
7	*p*-cymene	1018	1014	42.9	2.5	1.42	5.6	0.5	–	0	0	–	0	0	–	0.021
8	*o-c*ymene	1022	1009–1076	42.9	10.9	6.57	55.6	0.5	0.42	33.3	0.5	0.30	0	0	–	0.338
9	limonene	1023	10270	71.4	1.1	0.19	100	5.2	4.48	88.9	5.8	5.05	100	1.1	0.57	0.032
10	linalool	1090	1098	0	0	–	16.7	0.1	0.07	44.4	0.5	0.10	33.3	0.8	–	0.105
**11**	^1^ ***α*-campholenal**	1109	1105	85.7	1.3 a	0.58	100	4.1 b	1.45	100	3.6 b	1.61	66.7	3.1	1.35	0.004
**12**	***trans*-pinocarveol**	1135	1139	100	0.8 a	0.20	88.9	2.9 b	1.18	100	3.0 b	1.28	100	1.6	0.93	0.005
13	*trans*-sabinol	1139	1140	42.9	0.3	0.16	0	0	–	0	0	–	0	0	–	0.004
**14**	***trans*-verbenol**	1143	1144	100	2.9 a	1.12	100	13.1 b	5.39	100	13.6 b	5.79	100	6.6	3.43	0.001
**15**	***cis*-verbenol**	1145	1142	100	0.5 a	0.27	100	1.9 b	0.88	100	1.8 b	0.88	66.7	1.2	0.23	0.001
**16**	**pinocarvone**	1158	1160	85.7	0.4 a	0.04	100	1.6 b	0.50	88.9	1.8 b	0.62	66.7	1.4	0.34	0.003
17	*p*-mentha-1,5-dien-8-ol	1164	1166	0	0	–	5.6	0.4	0.31	44.4	0.7	0.39	0	0	–	0.016
**18**	***α*-phellandrene-8-ol**	1165	1166	0	0 a	–	77.8	0.9 b	0.32	0	0 a	–	0	0	–	<0.001
19	terpinen-4-ol	1174	1175	42.9	1.0	0.51	16.7	0.3	0.03	22.2	0.4	0.06	0	0	–	0.241
**20**	**myrtenal**	1192	1193	85.7	0.5 a	0.13	100	1.9 b	0.63	88.9	1.7 b	0.57	66.7	1.2	0.24	0.001
**21**	**myrtenol**	1193	1194	85.7	0.7 a	0.35	100	1.5 b	0.63	88.9	1.3	0.54	33.3	1.1	–	0.014
**22**	**verbenone**	1205	1205	42.9	0.4 a	0.29	100	2.7 b	1.19	100	2.7 b	1.38	100	1.2	0.67	<0.001
**23**	***trans*-carveol**	1219	1215	14.3	0.6 a	–	94.4	1.9 b	0.69	88.9	1.6	0.60	33.3	0.8	–	<0.003
**24**	**carvone**	1241	1242	0	0 a	–	94.4	1.3 b	0.18	77.8	1.1	0.52	0	0	–	<0.001
25	bornyl acetate	1287	1285	100	10.4	4.89	100	11.9	4.74	100	9.3	5.73	100	4.1	2.81	0.098
**26**	***α*-cubebene**	1347	1345	0	0	–	0	0 a	–	0	0	–	100	1.0 b	0.40	<0.001
**27**	***α*-copaene**	1372	1376	0	0	–	11.1	0.2 a	0.01	0	0	–	100	2.6 b	1.34	<0.001
**28**	***β*-cubebene**	1380	1389	0	0 a	–	77.8	4.9 b	3.21	11.1	6.0 a	0.04	0	0	–	<0.001
29	*β*-elemene	1389	1391	42.9	0.3	0.08	55.6	0.8	0.46	55.6	0.7	0.28	0	0	–	0.243
30	*(E)-β*-caryophyllene	1414	1419	71.4	0.9	0.31	88.9	0.6	0.16	44.4	0.8	0.44	66.7	0.7	0.04	0.425
**31**	***β*-copaene**	1424	1432	14.3	0.4	–	22.2	0.2 a	0.13	33.3	3.6	3.80	100	12.2	8.15	0.006
32	*α-h*umulene	1448	1452	42.9	0.4	0.04	38.9	0.2	0.21	22.2	1.3	1.34	0	0	–	0.588
**33**	***γ*-muurolene**	1475	1477	100	0.8 a	0.23	0	0 b	–	0	0 b	–	33.3	0.7	–	<0.001
**34**	**germacrene D**	1480	1480	100	11.2 a	4.80	22.2	8.1 b	4.21	88.9	2.5	1.65	100	1.5	0.20	<0.001
35	*β*-selinene	1481	1486	57.1	0.4	0.01	16.7	0.3	0.08	22.2	0.7	0.01	0	0	–	0.155
36	*epi*-cubebol	1489	1493	14.3	0.7	–	0	0	–	11.1	1.0	–	66.7	1.6	1.24	0.007
**37**	***α*-muurolene**	1499	1499	28.6	0.4	0.03	0	0 a	–	22.2	1.1	0.16	100	1.4 b	0.78	<0.001
**38**	***γ*-cadinene**	1507	1513	85.7	0.7 a	0.26	16.7	0.4 b	0.09	0	0 b	–	0	0	–	<0.001
**39**	**cubebol**	1514	1515	0	0	–	0	0 a	–	22.2	1.5	0.04	100	2.8 b	2.08	<0.001
**40**	***δ*-cadinene**	1520	1524	14.3	0.5	–	0	0 a	–	22.2	1.1	0.16	100	1.3 b	0.90	<0.001
41	epoxyazulene	1561	1554	71.4	6.4	2.98	55.6	1.9	1.22	88.9	1.6	1.35	0	0	–	0.054
42	*(E)-n*erolidol	1565	1564	71.4	0.7	0.28	11.1	0.4	0.13	11.1	0.4	–	0	0	–	0.002
**43**	**spathulenol**	1575	1576	100	5.0 a	2.97	38.9	0.9 b	0.74	66.7	2.8	3.96	100	1.3	0.20	<0.001
44	caryophyllene oxide	1578	1581	100	3.6	2.44	100	3.6	2.17	100	8.4	8.26	100	8.5	2.48	0.083
45	viridiflorol	1586	1590	0	0	–	0	0	–	0	0	–	33.3	1.2	–	0.010
**46**	**humulene epoxide II**	1607	1606	0	0	–	0	0 a	–	77.8	4.5 b	8.60	0	0	–	<0.001
**47**	**isospathulenol**	1627	1630	100	6.1 a	6.47	83.3	2.3	1.52	66.7	2.1	0.73	33.3	1.3 b	–	0.018
	Monoterpene hydrocarbons			100	25.4	16.52	100	28.0	20.88	100	26.4	18.09	100	35.9	11.86	0.608
	Oxygenated monoterpenes			100	18.5 a	6.04	100	46.6 b	15.42	100	41.5 b	16.35	100	19.2	7.60	<0.001
	Sesquiterpene hydrocarbons			100	15.3 b	6.63	100	7.9	6.00	100	6.0	4.85	100	20.7 a	10.64	0.007
	Oxygenated sesquiterpenes			100	19.7 a	8.85	100	7.1 b	3.48	100	17.0	15.54	100	14.7	5.25	<0.001
	Oxygenated diterpenes			14.3	0.1	–	11.1	0.9	0.19	22.2	1.0	0.51	0	0	–	0.479

^1^ Compounds in bold were included in the PCA. Compounds are listed in order of their linear retention indices (LRI exp.) calculated using homologous series of *n*-alkanes (C8–C26). LRI ref.—linear retention indices from NIST 14 data basis and reference. The mean values of the compounds marked with the letters (a, b) differ significantly at *p* ≤ 0.05 between species according to the Kruskal–Wallis test. *n*—number of distilled accessions per species.

**Table 2:** The frequency of distribution (F, %) of the compounds detected in more than 30% of the leaf EO samples in at least one of the four *Solidago* species; their mean relative percentage (M) with SD and significance (*p*) of interspecies differences according to the Kruskal–Wallis two-tailed test.

No	Compounds	^a^ LRI Exp.	^b^ LRI Ref.	*S. gigantea* (*n* = 7)			*S. canadensis* (*n* = 18)			*S. × niederederi* (*n* = 9)			*S. virgaurea* (*n* = 3)			*p*
				F	M	SD	F	M	SD	F	M	SD	F	M	SD	
**1**	***α*-pinene**	930	930	100	7.8	2.18	100	8.6	5.69	100	11.9	10.37	100	16.0	11.77	0.856
**2**	**^1^ camphene**	945	948	100	2.8 a	0.76	100	1.2 b	0.40	77.8	1.1 b	0.45	66.7	0.5 b	0.07	<0.001
3	thuja-2,4(10)-diene	950	957	0	0	–	11.1	0.2	0.13	33.3	0.6	0.13	33.3	0.3	–	0.187
4	sabinene	971	976	42.9	1.1	0.61	44.4	0.6	0.42	33.3	1.1	1.21	66.7	0.3	0.01	0.926
5	*β*-pinene	974	980	100	2.2	0.88	94.4	1.4	0.61	88.9	1.9	1.16	66.7	3.5	0.64	0.193
6	*β*-myrcene	992	988	28.6	1.2	0.12	5.6	1.0	–	22.2	0.8	0.49	33.3	0.4	–	0.372
**7**	***o-c*ymene**	1022	1009–1076	100	2.1 a	1.40	50.0	0.5 b	0.46	22.2	0.4 b	0.06	0	0 b	–	<0.001
**8**	**limonene**	1023	1027	100	1.1 a	0.16	100	2.5 b	2.17	100	2.2	1.56	66.7	1.1	0.35	0.006
**9**	***α*-campholenal**	1109	1105	14.3	0.6 a	–	77.8	0.8	0.72	77.8	2.6	1.68	100	2.8 b	0.52	0.007
**10**	***trans*-pinocarveol**	1135	1139	42.9	0.3 a	0.16	66.7	0.9 a	0.50	77.8	1.6	0.75	100	3.6 b	1.83	0.003
**11**	***trans*-verbenol**	1143	1144	42.9	0.5 a	0.23	100	2.7 b	1.81	100	8.9 b	6.85	100	24.6 b	21.21	<0.001
**12**	***cis*-verbenol**	1145	1142	0	0 a	–	27.8	0.4	0.27	44.4	1.6	0.39	100	1.5 b	0.82	0.004
**13**	**pinocarvone**	1158	1160	0	0 a	–	22.2	0.6 a	0.31	66.7	1.1	0.51	100	1.8 b	0.04	<0.001
14	myrtenal	1192	1193	0	0	–	72.2	0.6	0.29	55.6	1.6	0.48	66.7	1.6	0.02	0.024
15	myrtenol	1193	1194	0	0	–	55.6	0.3	0.25	55.6	1.0	0.33	66.7	1.2	0.03	0.038
**16**	**verbenone**	1205	1206	42.9	0.3 a	0.16	88.9	1.5	1.35	77.8	5.2 b	6.77	100	7.3 b	8.37	0.008
17	*cis*-carveol	1208	1206	0	0	–	5.6	1.1	–	0	0	–	33.3	0.7	–	0.168
18	*trans*-carveol	1219	1215	0	0	–	16.7	0.2	0.07	44.4	0.9	0.21	33.3	0.8	–	0.066
**19**	**bornyl acetate**	1287	1285	100	22.4 a	5.37	100	18.7 a	7.55	100	14.0	5.53	100	2.6 b	0.46	0.004
20	thymol	1295	1297	0	0	–	5.6	0.5	–	11.1	9.5	–	33.3	3.8	–	0.330
21	carvacrol	1306	1308	0	0	–	11.1	23.3	0.63	11.1	6.4	–	66.7	3.2	0.42	0.079
22	*α*-copaene	1372	1376	0	0	–	22.2	0.3	0.09	0	0	–	66.7	0.5	0.03	0.059
23	*β*-bourbonene	1378	1385	85.7	0.9	0.45	50.0	0.5	0.46	22.2	2.0	2.27	33.3	1.8	–	0.056
24	*β*-cubebene	1380	1389	57.1	14.1	8.41	38.9	21.3	10.25	22.2	20.1	5.25	0	0	–	0.467
**25**	***β*-elemene**	1389	1391	28.6	0.8 a	0.04	83.3	1.7 b	1.45	66.7	0.8	0.57	33.3	0.4	–	0.005
26	*(E)-β*-caryophyllene	1414	1419	85.7	1.0	0.27	61.1	2.5	2.14	66.7	2.0	1.67	66.7	1.5	1.42	0.985
27	*β*-copaene	1424	1432	14.3	0.5	–	50.0	0.6	0.19	22.2	3.8	4.56	66.7	3.2	0.79	0.161
28	*α*-humulene	1448	1452	42.9	0.5	0.08	61.1	0.8	0.54	33.3	1.9	1.89	33.3	0.4	–	0.380
**29**	***γ*-muurolene**	1475	1477	100	0.8 a	0.23	0	0 b	–	0	0 b	–	33.3	0.7	–	<0.001
30	germacrene D	1480	1480	42.9	5.9	5.95	50.0	19.6	15.22	66.7	8.8	5.22	66.7	5.2	6.46	0.751
31	*β*-selinene	1481	1486	14.3	0.6	–	38.9	0.5	0.47	0	0	–	0	0	–	0.103
32	*epi*-cubebol	1489	1493	0	0	–	0	0	–	0	0	–	66.7	1.4	0.31	0.012
33	*α*-muurolene	1499	1499	0	0	–	0	0	–	0	0	–	33.3	0.3	–	0.222
34	*β*-bisabolene	1505	1509	0	0	–	11.1	5.0	1.05	11.1	4.7	–	33.3	1.8	–	0.569
35	*γ*-cadinene	1507	1513	100	1.0	0.32	5.6	0.7	–	0	0	–	0	0	–	<0.001
36	cubebol	1514	1515	0	0	–	0	0	–	0	0	–	66.7	0.7	0.10	0.014
37	*δ*-cadinene	1520	1524	0	0	–	5.6	0.7	–	11.1	0.9	–	33.3	0.4	–	0.384
**38**	**epoxyazulene**	1561	1554	100	6.1 a	3.22	100	2.7	1.43	66.7	2.9 b	2.49	33.3	0.3 b	–	0.001
**39**	***(E)*-nerolidol**	1565	1564	85.7	0.9 a	0.20	38.9	0.6	0.20	0	0 b	–	0	0	–	0.001
**40**	**spathulenol**	1575	1576	100	6.7 a	6.25	88.9	1.2 b	0.98	66.7	2.1 b	1.94	100	3.1	1.30	0.003
**41**	**caryophyllene oxide**	1578	1581	100	3.1 a	0.77	77.8	3.9 a	2.24	100	10.4 b	11.18	100	9.0	6.36	0.004
42	humulene epoxide II	1607	1606	57.1	2.4	1.26	38.9	2.7	1.04	22.2	3.2	0.92	66.7	5.0	1.44	0.276
**43**	**isospathulenol**	1627	1630	100	6.3 a	3.11	100	4.8 a	2.54	66.7	3.7	2.26	0	0 b	–	0.004
	Monoterpene hydrocarbons			100	16.6	4.08	100	14.4	9.42	100	17.7	10.92	100	20.1	15.28	0.634
	Oxygenated monoterpenes			100	23.1	5.64	100	27.9	11.2	100	35.7	13.07	100	49.0	27.81	0.072
	Sesquiterpene hydrocarbons			100	14.9	7.85	94.4	23.3	15.3	100	15.0	11.32	66.7	8.6	10.93	0.252
	Oxygenated sesquiterpenes			100	23.3	10.03	100	13.3	6.07	100	16.9	12.56	100	16.9	7.63	0.181
	Oxygenated diterpenes			0	0	–	5.6	0.7	0.26	11.1	0.1	0.33	0	0	–	0.375

^1^ Compounds in bold were included in the PCA. Compounds are listed in order of their linear retention indices (LRI exp.) calculated using homologous series of *n*-alkanes (C8–C26). LRI ref.—linear retention indices from NIST 14 data basis and reference. The mean values of the compounds marked with the letters (a, b) differ significantly at *p* ≤ 0.05 between species according to the Kruskal–Wallis test. *n*—number of distilled accessions per species.

**Table 3:** Collection sites data on *Solidago gigantea* (SG), *S. canadensis* (SC), *S. × niederederi* (SN), and *S. virgaurea* (SV).

Collection Site	Altitude, m	Latitude N	Longitude E	Number of Accessions			
				SG	SC	SN	SV
Pavilnys, Vilnius distr.	215	54°40′35″	25°23′01″	3	3	1	–
Didieji Pupojai, Vilnius	206	54°42′38″	25°23′29″	1	3	3	2
Rokantiškės, Vilnius	203	54°40′03″	25°22′58″	3	2	1	–
Raudondvaris, Vilnius distr.	149	54°52′32″	25°31′08″	–	4	–	–
Dvariškės, Vilnius distr.	149	54°49′17″	25°16′23″	–	3	3	1
Karklinė, Vilnius distr.	164	54°54′26″	25°33′40″	–	3	1	–

**Table 4:** Repeatability and intermediate precision on the relative retention time (t_R_,) and peak area (A) of the five analytes in *S. canadensis* EOs expressed as relative standard deviation (RSD, %).

Analytes	Repeatability (Run-to-Run)				Intermediate Precision (Day-to-Day)			
	t_R_, min	RSD, %	A, %	RSD, %	t_R_, min	RSD, %	A, %	RSD, %
*α*-pinene	11.85	0.23	24.57	0.45	11.92	0.82	24.83	1.51
limonene	15.24	0.29	9.07	0.33	15.31	0.73	9.17	1.42
*trans*-verbenol	23.20	0.22	10.36	0.41	23.36	0.92	10.54	2.21
bornyl acetate	32.70	0.32	10.21	0.43	32.77	0.54	10.26	0.98
*β*-cubebene	44.59	0.26	2.82	0.38	44.74	0.60	2.84	1.02

## Data Availability

All data generated during this study are included in this article.
